# Common midwife toad ranaviruses replicate first in the oral cavity of smooth newts (*Lissotriton vulgaris*) and show distinct strain-associated pathogenicity

**DOI:** 10.1038/s41598-019-41214-0

**Published:** 2019-03-14

**Authors:** Bernardo Saucedo, Trenton W. J. Garner, Natasja Kruithof, Steven J. R. Allain, Mark J. Goodman, Raymond J. Cranfield, Chris Sergeant, Diego A. Vergara, Marja J. L. Kik, María J. Forzán, Steven J. van Beurden, Andrea Gröne

**Affiliations:** 1 Utrecht University, Utrecht, the Netherlands; 2 Dutch Wildlife Health Centre, Utrecht, the Netherlands; 3 Zoological Society of London, London, United Kingdom; 4 Durrell Institute of Ecology and Evolution, University of Kent, London, UK; 5 Cambridgeshire and Peterborough Amphibian and Reptile Group, London, United Kingdom; 6 Essex Amphibian and Reptile Group, London, United Kingdom; 7 National Autonomous University of, Mexico, Mexico; 8 Cornell University, College of Veterinary Medicine USA, Ithaca, USA

## Abstract

*Ranavirus* is the second most common infectious cause of amphibian mortality. These viruses affect caudates, an order in which information regarding *Ranavirus* pathogenesis is scarce. In the Netherlands, two strains (CMTV-NL I and III) were suspected to possess distinct pathogenicity based on field data. To investigate susceptibility and disease progression in urodeles and determine differences in pathogenicity between strains, 45 adult smooth newts (*Lissotriton vulgaris*) were challenged via bath exposure with these ranaviruses and their detection in organs and feces followed over time by PCR, immunohistochemistry and *in situ* hybridization. *Ranavirus* was first detected at 3 days post infection (p.i.) in the oral cavity and upper respiratory mucosa. At 6 days p.i, virus was found in connective tissues and vasculature of the gastrointestinal tract. Finally, from 9 days p.i onwards there was widespread *Ranavirus* disease in various organs including skin, kidneys and gonads. Higher pathogenicity of the CMTV-NL I strain was confirmed by higher correlation coefficient of experimental group and mortality of challenged animals. *Ranavirus*-exposed smooth newts shed virus in feces intermittently and infection was seen in the absence of lesions or clinical signs, indicating that this species can harbor subclinical infections and potentially serve as disease reservoirs.

## Introduction

The common midwife toad virus (CMTV) is a *Ranavirus* (family *Iridoviridae*), initially reported in Spain in 2008^1^. *Ranaviruses* clustering genetically within the common midwife toad clade were subsequently recorded as the cause of mortality events involving various amphibian and reptile species throughout Europe^2–5^ and Asia^6,7^.

In the north of the Netherlands (NL), CMTV was the cause of an amphibian mass mortality event in 2010 at Dwingelderveld National Park (DNP)^8,9^. Since then, over half of the amphibian species native to the Netherlands have been affected by CMTV ranaviruses; particularly water frogs (*Pelophylax kl. esculentus*) and smooth newts (*Lissotriton vulgaris*)^10^. Macroscopic lesions consistently observed across affected amphibian host species included skin hemorrhages and edema. Histology revealed widespread necrosis and the presence of intracytoplasmic inclusion bodies^8^.

Molecular tests revealed that in addition to the northern CMTV *Ranavirus* strain, two other phylogenetically distinct CMTV *Ranavirus* strains occurred in the east and south of the Netherlands. Dutch *Ranavirus* strains were designated as CMTV-NL I, II and III^11^. Based on gene truncations, field mortality patterns and changes in animal abundance after mortality events, the strain from the north (CMTV-NL I) appeared to be more pathogenic phenotype than the southern strain (CMTV-NL III). From this, it was previously concluded that CMTV NL-I, the more virulent strain, was recently introduced into the Netherlands and that CMTV NL-III was potentially endemic with attenuated virulence^10,11^.

The other Dutch strain (CMTV-NL II) was obtained from a pond within a private property in the centre-east of the country. No field data was available for this strain. CMTV NL-II was not included in this experiment.

The smooth newt is one of the amphibians commonly affected by CMTV-NL ranaviruses. Juvenile smooth newts can quickly colonize new habitats and disperse hundreds of meters from breeding to overwintering sites within a year^12^. They are often found in the same waterbodies as water frogs (*Pelophylax* spp.) which have suffered population declines due to *Ranavirus* infection^10^. All of these characteristics make them potential *Ranavirus* hosts, and if so, potential spreading vectors. Subclinical *Ranavirus* infections have been recorded in other caudate species: adult tiger salamanders introduce *Ambystoma tigrinum* virus (ATV) into waterbodies when returning to breeding sites after hibernation or when they are used as bait^13,14^.

Recent research on *Ranavirus* pathogenesis, tissue tropism, and dose-dependent susceptibility has focused on anurans^15^. In contrast, most of the experimental work regarding caudates is limited primarily to distinct aspects of *Ranavirus* strain pathogenicity or the influence of environmental factors on the development of disease^16^.

In order to study the susceptibility of smooth newts to CMTV-NL *Ranavirus* disease, to determine whether CMTV NL *Ranavirus* strains differ in pathogenicity, and to find out whether this species can harbor subclinical infections and excrete virus, smooth newts were challenged with Dutch CMTV isolates. The smooth newt was chosen as an experimental model due to field data on its susceptibility to CMTV-NL^10^. Through this experiment, the pathogenesis and fecal shedding of CMTV-NL ranaviruses were studied by tracing the virus within the tissues and feces of the hosts over time. The pathogenicity of CMTV-NL strains I and III was compared by characterizing lesion severity through semi-quantitative scoring.

## Results

### Smooth newts are susceptible to infection with both CMTV-NL *Ranavirus* strains

**Figure 1:**
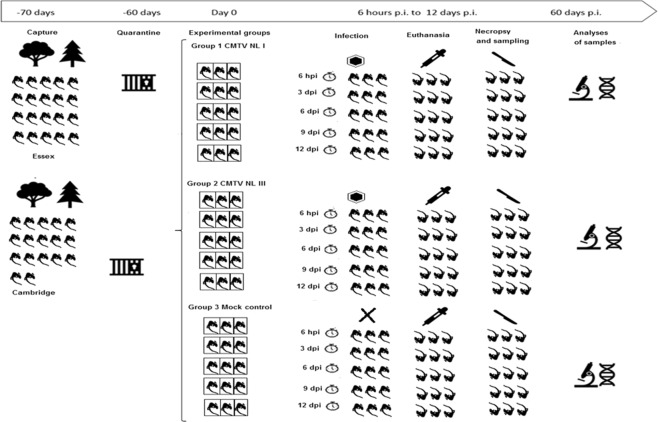
Experimental set-up: A total of 45 smooth newts were first captured in Essex and Cambridge at sites with no previous history of *Ranavirus* infection. Afterwards they were transported to the facilities of IoZ and kept under quarantine for 2 months. After completion of the quarantine period the animals were divided into 3 groups of 15 animals and assigned three treatments (CMTV-NL *Ranavirus* I, CMTV-NL *Ranavirus* III and cell culture medium for the control group). The 3 groups were subdivided into 5 groups of 3 animals which were euthanized at 5 different p.i. time points (6 hours, 3, 6, 9 and 12 days p.i.). Thereafter, necropsy was performed and organ samples were collected and preserved in 10% formalin and 70% alcohol for histopathological and PCR analyses respectively. This figure was designed and drawn by the first author of the paper.

Adults newts captured in the wild were challenged with two CMTV-NL strains or medium and euthanized at 5 post-infection (p.i.) time points to study disease progression. The experimental set up is schematically illustrated in Fig. 1.

Smooth newts in all experimental groups had experienced a minor loss of weight by the end of the trial, but weight loss did not vary by treatment (Supplementary Table S1.; ANOVA; *p* = 0.6). All animals from the control group remained healthy until the end of the experiment.

**Figure 2:**
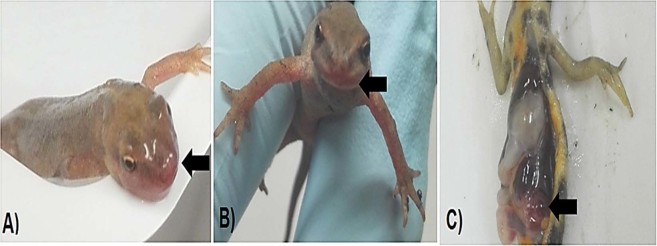
Macroscopic lesions: (**A**,**B**) CMTV-NL I *Ranavirus*-infected animals showing moderate erythema and hemorrhage of the mouth region (arrow), (**C**) Necropsy of smooth newt at 12 days p.i. Macroscopic changes include edema expanding the abdominal fatty tissue and hemorrhage of viscera within the coelomic cavity (arrow).

Three of the CMTV-NL I-exposed animals had mild erythema and/or hemorrhages in the mouth and gular region, lesions were evident from day 9 p.i. and persisted until the end of the experiment (Fig. 2), while only one CMTV-NL III-exposed animal exhibited mouth erythema at day 7 p.i. Sudden death without clinical signs occurred in three CMTV-NL I-exposed newts, at days 7, 9 and 10 p.i., while none of the CMTV-NL III-exposed animals died or reached humane end-points before the end of the experiment.

### *Ranavirus* first replicates in the upper alimentary tract of smooth newts

**Figure 3:**
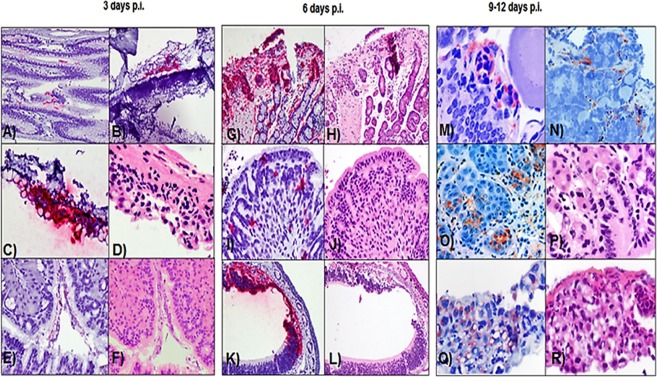
CMTV NL I *Ranavirus* detection over time: (**A**) ISH viral RNA staining in the esophageal lumen (**B**) ISH viral RNA staining in the upper respiratory tract mucosa (**C**) ISH viral RNA staining in the oral mucosa, (**D**) Serial section of oral mucosa displaying mild lymphocytic infiltrates in the submucosa. Hematoxylin and Eosine. (**E**) ISH viral RNA staining in the gastric lumen. (**F**) Serial section of stomach without microscopic alterations. (**H** and **E**,**G**) Marked positive ISH staining in the oral mucosa. (**H**) Serial section of the oral mucosa, Hematoxylin and Eosine. (**I**) Moderate positive ISH staining of the gastric mucosa. (**J**) Serial section of the stomach. Hematoxylin and Eosine. (**K**) Moderate positive ISH staining of the upper respiratory mucosa. L) Serial sections of upper respiratory mucosa. (**M**) 9 days p.i., Moderate IHC signal is present in the ovary (**N**) 9 days p.i., Moderate IHC signal in the connective tissues surrounding the testes (**O**) 9 days p.i., Marked IHC signal in the mucosal glands of the stomach P) Serial section of the stomach displaying sloughing of the necrotic cells (**Q**) 12 days p.i. Marked IHC signal in the epidermis. (**R**) Serial section of the skin presenting with marked spongiosis of the epidermis and exocytosis.

All smooth newt tissues sampled at 6 hours p.i. tested negative for infection using *in situ* hybridization ISH. However, viral RNA was first detected at 3 days p.i. by ISH staining in the upper respiratory tract mucosa, oral mucosa, and stomach contents of a CMTV-NL I-infected animal. (Fig. 3). ISH showed evidence of infection in multiple tissues at 6 and 9 days p.i.,(Table 1).

**Table 1:** ISH, IHC, and PCR results of *Ranavirus*-infected animals.

CMTV-NL I	6 h p.i.			3 d p.i.			6 d p.i.			9 d p.i.			12 d p.i.		
	ISH	IHC	PCR	ISH	IHC	PCR	ISH	IHC	PCR	ISH	IHC	PCR	ISH	IHC	PCR
Mouth	1 ND,2−	3 −	3 ND	1ND,1+,1−	3−	3 ND	2 ND, 1+	1+,2−	3 ND	3 ND	1+,2−	3 ND	3 ND	1+,2−	3 ND
URT	1 ND,2−	3 −	3 ND	1ND,1+,1−	3−	3 ND	3 ND	1+,2−	3 ND	3 ND	1+,2−	3 ND	3 ND	3+	3 ND
Esophagus	1 ND,2−	3 −	3 ND	1ND,1− *	3−	3 ND	2 ND,1+	1+,2−	3 ND	2 ND,1+	1+,2−	3 ND	3 ND	3−	3 ND
Stomach	1 ND,2−	3 −	3 ND	1ND,1− *	3−	3 ND	2 ND,1+	1+,2−	3 ND	2 ND,1+	2+,1−	3 ND	3 ND	2+,1−	3 ND
Spleen	1 ND,2−	3 −	3 ND	1ND,2−	3−	3 ND	3 ND	1+,2−	3 ND	3 ND	1 ND,2−	3 ND	3 ND	1ND, 2−	3 ND
Kidney	1 ND,2−	3 −	3 ND	1ND,2−	3−	3 ND	3 ND	1ND,2−	3 ND	2 ND, 1+	2+,1−	3 ND	3 ND	3−	3 ND
Gonad	1 ND,2−	3 −	3 ND	1ND,2−	3−	3 ND	2 ND,1+	1+,2−	3 ND	2 ND, 1+	2+,1−	3 ND	3 ND	1+,2−	3 ND
Pancreas	1 ND,2−	3 −	3 ND	1ND,2−	3−	3 ND	3 ND	3−	3 ND	2 ND, 1+	3+	3 ND	3 ND	3−	3 ND
Heart	1 ND,2−	3 −	3 ND	1ND,2−	3−	3 ND	2 ND,1+	1+,2−	3 ND	2 ND, 1+	1+,2−	3 ND	3 ND	3−	3 ND
Liver	1 ND,2−	3 −	3 −	1ND,2−	3−	1+,2−	2 ND,1+	1+,2−	2+,1−	2 ND, 1+	3+	3+	3 ND	3+	3+
Intestine	1 ND,2−	3 −	3+	1ND,2−	3−	2+,1−	2 ND,1+	3−	2+,1−	2 ND, 1+	3+	3+	3 ND	3+	3+
Skin	1 ND,2−	3 −	3 −	1ND,2−	3−	3−	2 ND,1+	3−	3−	3 ND	3+	3+	3 ND	3+	3+
**CMTV-NL III**															
Mouth	1 ND,2−	3 −	3 ND	1ND,2−	3−	3 ND	2 ND, 1+	1+,2−	3 ND	3 ND	3−	3 ND	3 ND	1+,2−	3 ND
URT	1 ND,2−	3 −	3 ND	1ND,2−	3−	3 ND	3 ND	1+,2−	3 ND	3 ND	3−	3 ND	3 ND	1+,2−	3 ND
Esophagus	1 ND,2−	3 −	3 ND	1ND,2−	3−	3 ND	3 ND	1+,2−	3 ND	2 ND,1+	1+,2−	3 ND	3 ND	3−	3 ND
Stomach	1 ND,2−	3 −	3 ND	1ND,2−	3−	3 ND	2 ND, 1+	3−	3 ND	2 ND,1+	3−	3 ND	3 ND	1+,2−	3 ND
Spleen	1 ND,2−	3 −	3 ND	1ND,2−	3−	3 ND	3 ND	1ND,2−	3 ND	3 ND	3−	3 ND	3 ND	3−	3 ND
Kidney	1 ND,2−	3 −	3 ND	1ND,2−	3−	3 ND	2 ND, 1+	3−	3 ND	2 ND,1+	1+,2−	3 ND	3 ND	3−	3 ND
Gonad	1 ND,2−	3 −	3 ND	1ND,2−	3−	3 ND	2 ND, 1+	3−	3 ND	2 ND,1+	2+,1−	3 ND	3 ND	1+,2−	3 ND
Pancreas	1 ND,2−	3 −	3 ND	1ND,2−	3−	3 ND	2 ND, 1+	3−	3 ND	2 ND,1+	3−	3 ND	3 ND	3−	3 ND
Heart	1 ND,2−	3 −	3 ND	1ND,2−	3−	3 ND	2 ND, 1+	3−	3 ND	2 ND,1+	1+,2−	3 ND	3 ND	3−	3 ND
Liver	1 ND,2−	3 −	3 −	1ND,2−	3−	2+,1−	2 ND, 1+	2+,1−	1+,2−	2 ND,1+	1+,2−	1+,2−	3 ND	1+,2−	2+,1−
Intestine	1 ND,2−	3 −	3+	1ND,2−	3−	3+	3 ND	3−	3+	2 ND,1+	1+,2−	3+	3 ND	1+,2−	1+,2−
Skin	1 ND,2−	3 −	3 −	1ND,2−	3−	3−	2 ND, 1+	3−	3−	3 ND	1+,2−	3−	3 ND	1+,2-	3−

Animals from the 6 hours p.i., 3 days p.i. and the control group tested negative for all tests in all organs.

Positive (+), negative (−), organ not done (ND) * signal present only in the lumen.

Positive immunohistochemistry (IHC) staining indicative of viral protein production was not observed until 6 days p.i., when mild single cell staining was detected in the liver, esophagus, oral mucosa and upper respiratory tract mucosa of both *Ranavirus*-exposed experimental groups. Positive staining also occurred in splenic macrophages, blood vessels of the heart and connective tissues of the stomach and gonads in CMTV-NL I-exposed animals. By day 9 p.i., an IHC signal was still present in the previously positive connective tissues of the reproductive tract and liver, and had spread to the skin, intestine, pancreas, and kidney in affected animals from both exposed groups. At day 12 p.i. viral proteins were still present in most tissues (upper respiratory and mouth mucosa, stomach, intestine, liver, connective tissues of the gonads and in the skin) of animals from both *Ranavirus*-infected groups (Fig. 3). However, viral proteins could no longer be detected in the kidney, spleen, heart and pancreas (Table 1). Virus protein production was not observed at any time points in the peripheral or central nervous system of CMTV-NL infected smooth newts.

**Figure 4:**
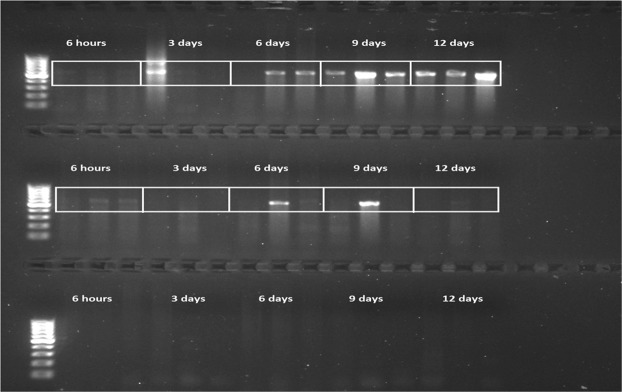
CMTV-NL *Ranavirus* PCR detection: *Ranavirus* detection over time by PCR of intestine. Each white rectangle encloses 3 replicates per time point. First top row shows detection of CMTV-NLI at selected time points. Bands of varying intensity are observed in all animals with the exception of one animal at 3 days and another one at 6 days p.i. The brightness of the bands intensifies over time. Second row shows bands in most CMTV-NLIII- infected animals. Third row shows no bands for *Ranavirus* in the tissues from the control group. The original gel photo is available as Supplementary Fig. S1 and the β-actin results are available in Supplementary Fig. S2.

None of the control animals tested *Ranavirus* positive at any of the time points using PCR and they all tested PCR-positive for the control gene β-actin (Supplementary Fig. S1). *Ranavirus* DNA was detected by PCR at the earliest time point (6 hours p.i.) in intestine samples of both *Ranavirus*-infected groups and in the skin of one CMTV-NL III-infected animal. At day 3 p.i., DNA was detected in the liver of animals from both *Ranavirus*-infected groups, while 6, 9 and 12 days p.i., DNA was present in the liver and intestine of both *Ranavirus*-infected groups, but only the skin of CMTV-NL I-exposed animals tested positive 9 and 12 days p.i. (Fig. 4, Table 1/Supplementary Fig. S2)

### Lesions are more severe in newts infected with CMTV-NL I

Pathogenicity of the CMTV-NL *Ranavirus* strains were evaluated through semi-quantitative scoring based on a previously established system^10^ which is detailed further in the Materials and Methods section.

**Figure 5:**
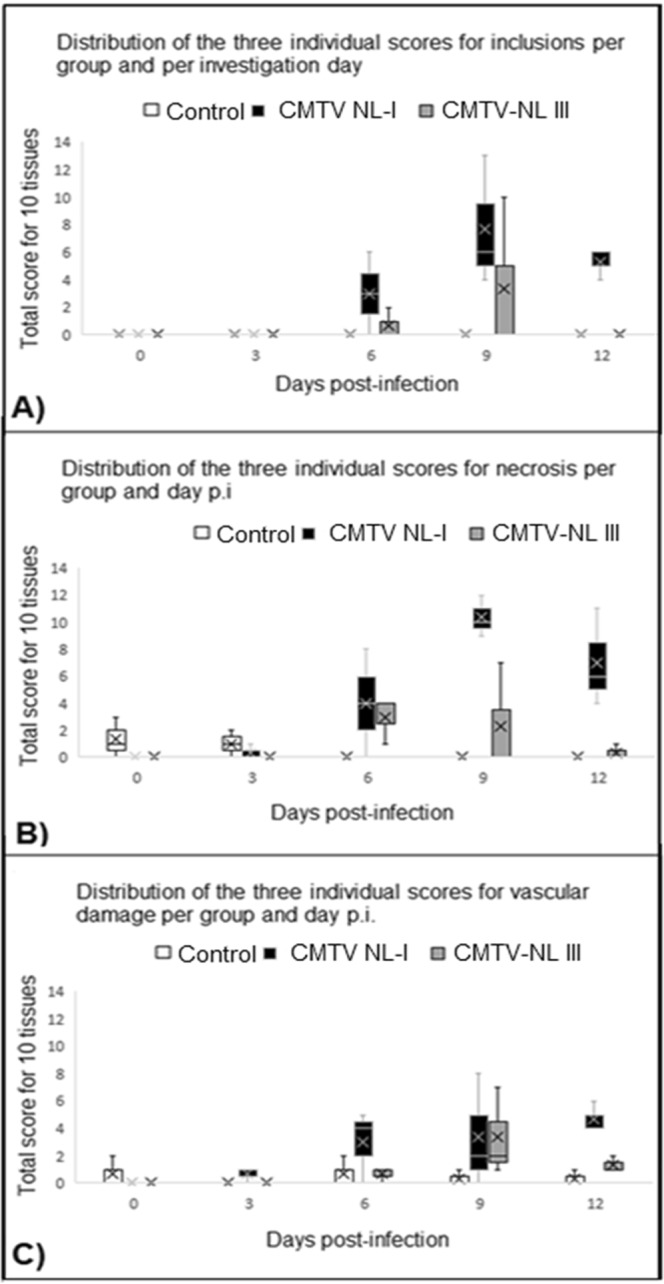
Lesion severity scoring, Box plot analysis of the severity of the three hallmark lesions of *Ranavirus* infection: Results on the semi-quantitative scoring of experimental groups show that the amount of inclusion bodies (**A**) increased over time in *Ranavirus* -infected animals especially at day 9 p.i., with the overall highest severity scores observed in CMTV-NL I-infected animals. None of the control animals had inclusion bodies in any of the organs. Similar to the amount of inclusion bodies, necrosis (**B**) in multiple tissues also increased over time reaching a peak at day 9 p.i. and was most prominent in CMTV-NL I-infected animals. Necrosis was lower in CMTV-NL III-infected animals and almost negligible in the control animals. Finally, vascular damage (**C**) also increased over time and was also highest in CMTV-NL I-infected animals at days 9 and 12 p.i., slightly lower CMTV-NL III-infected animals and almost negligible in the control animals.

**Figure 6:**
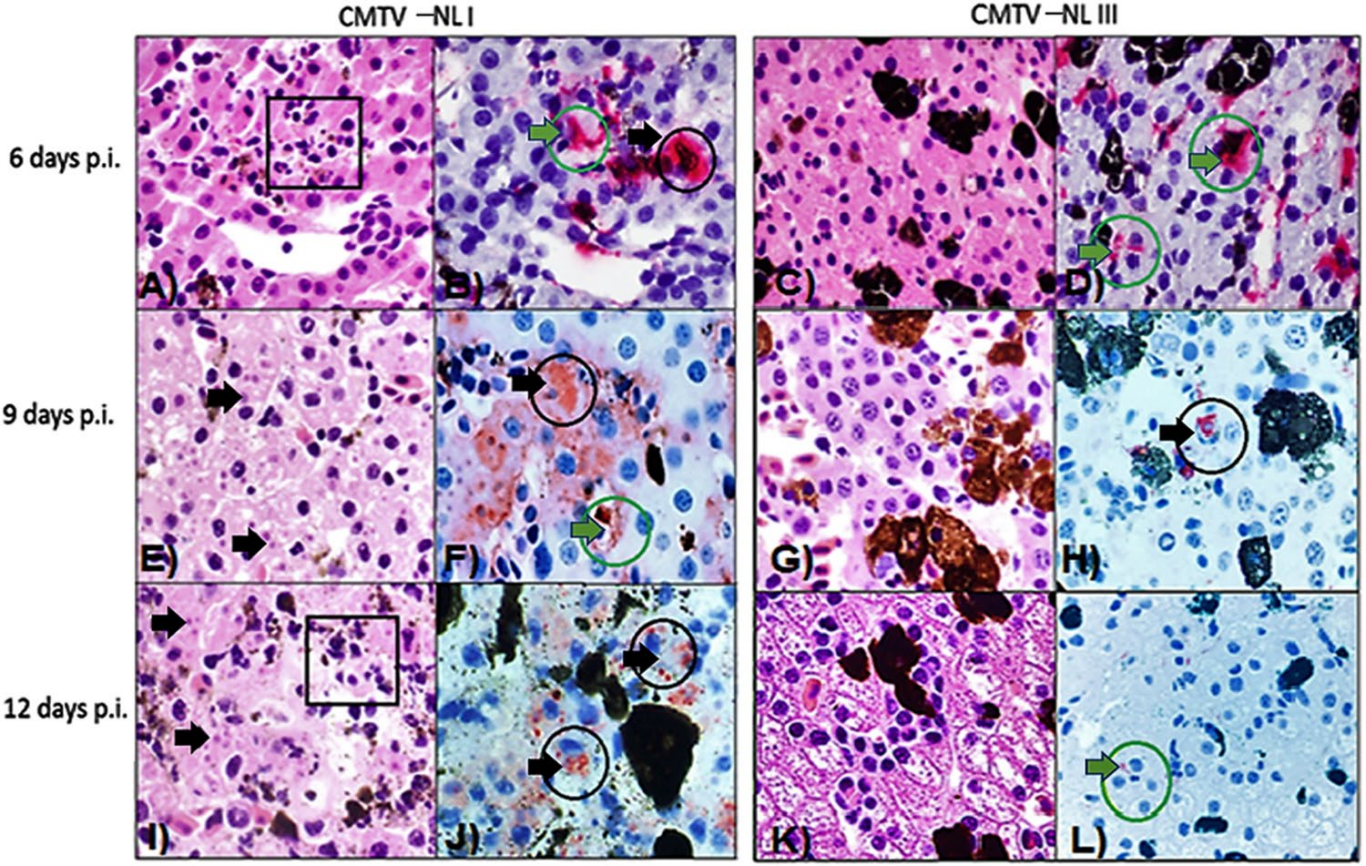
Comparison of lesion severity in the liver of smooth newts infected with CMTV-NL *Ranavirus* strains over time. (**A**,**B**) CMTV-NL I *Ranavirus*-infected smooth newt with a small area of necrosis (square) Hematoxylin and Eosine, and positive ISH immubolabelling of sinusoids (green arrow and circle) and hepatocytes (black arrow and circle). C and D) CMTV-NL III *Ranavirus*-infected smooth with no changes in Hematoxylin and Eosine, ISH immunolabelling restricted to sinusoids. (**E**,**F**) CMTV-NL I *Ranavirus*-infected smooth newt with numerous intracytoplasmic inclusion bodies (arrows) in hepatocytes, Hematoxylin and Eosine. Marked IHC immunolabelling of hepatocytes (black arrow and circle) and in sinusoids (green arrow and circle) (**G**,**H**) CMTV-NL III *Ranavirus*-infected smooth newt at 9 days p.i. with mild increase in the number of melano-macrophages, Hematoxylin and Eosine. Positive IHC immunolabelling is restricted to the cytoplasm of one hepatocyte (black arrow and circle). (**I**,**J**) CMTV-NL I *Ranavirus*-infected smooth newt with marked necrosis of the parenchyma (square) and rare presence of inclusion bodies (arrow) Hematoxylin and Eosine, IHC immunolabelling is observed within hepatocytes (black circles). K and L) CMTV-NL III *Ranavirus*-infected smooth newt, No microscopic abnormalities are seen with Hematoxylin and Eosine, IHC immunolabelling is scant and limited to a few sinusoids (green circle) IHC Anti-ECV polyclonal antibody, ISH FV3 probe.

Results showed that lesions were more severe in animals from Group 1 infected with CMTV-NL I *Ranavirus*, than in Group 2 animals infected with the CMTV-NL III *Ranavirus* (Fig. 5). In animals infected with CMTV-NL I *Ranavirus*, inclusions, necrosis and vascular damage were consistently present at days 9 and 12. The only consistent finding in animals infected with CMTV-NL III *Ranavirus* on those days was vascular damage. In the liver of CMTV-NL I *Ranavirus*-infected animals, IHC positive signal increased over time along with the evidence of necrosis and inclusions bodies. These changes were observed in the cytoplasm of hepatocytes and in the sinusoids. In contrast the IHC positive signal in CMTV-NL III *Ranavirus* infected animals remained scarce, mostly limited to sinusoids. Histopathology revealed an increase in the number of melano-macrophages, but necrosis and inclusion bodies were rarely observed (Fig. 6).

The Spearman rank correlation method was used to evaluate the lesion severity scores. It revealed high correlation coefficients among the lesion scores in CMTV-NL I-infected animals (all > 0.70; Supplementary Table S2); however, due to multiple ties, it was not possible to calculate reliable p-values (Supplementary Table S2).

No inclusion bodies were observed in animals from the control group. The results of the semi-quantitative scoring are available in Supplementary Table S3.

### Smooth newts infected with CMTV-NL-I *Ranavirus* shed virus intermittently through the feces

**Figure 7:**
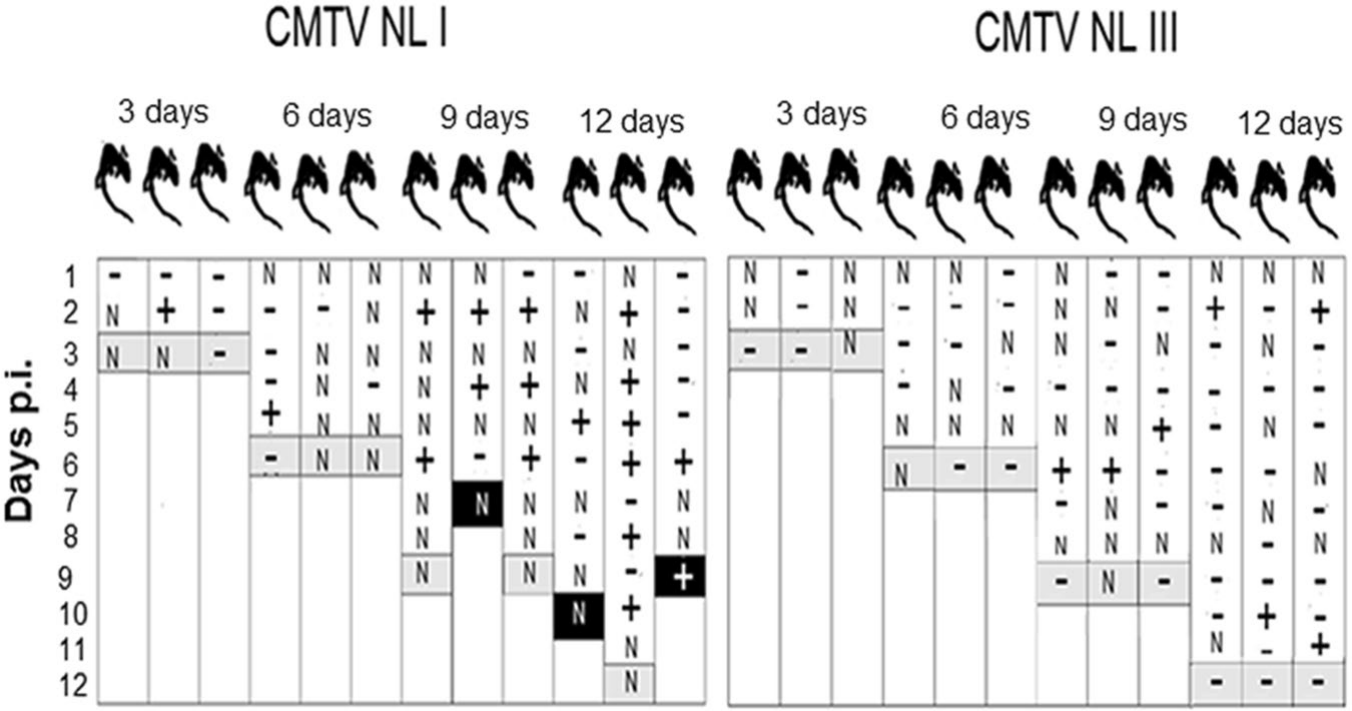
PCR detection of *Ranavirus* in fecal samples of animals from CMTV-NL I and CMTV-NL III *Ranavirus*-infected animals: The X axis shows the p.i. time point of euthanasia, while the Y axis shows the days p.i. Positive *Ranavirus* detection is represented by + sign while negative fecal samples are represented by a − sign. Grey bands represent the day at which the animals were euthanized, while the black bands represent days at which infected animals died prior to reaching the set time point of euthanasia. The letter “N” stands for no feces found. Control animals always tested negative for *Ranavirus* in fecal samples.

*Ranavirus* DNA was found in the feces of both CMTV-NL *Ranavirus*-infected animals at day 2 p.i. At day 3 p.i., none of the feces tested positive for *Ranavirus* DNA. Feces of CMTV-NL I-exposed animals tested positive intermittently and in a few animals repeatedly from day 4 to 10 p.i. In contrast, feces from CMTV-NL III-exposed animals tested positive only once from day 5 to 11 p.i. (Fig. 7). Control animals always tested PCR negative for *Ranavirus*.

## Discussion

The aims of this study were to determine the susceptibility of smooth newts to CMTV-NL *Ranavirus* infection, the pathogenesis of *Ranavirus* disease in these hosts, the difference in pathogenicity between two closely related Dutch CMTV *Ranavirus* strains and the ability of smooth newts to excrete *Ranavirus* and harbor subclinical infections.

Pathology, PCR, ISH and IHC, all illustrate how smooth newts are susceptible to CMTV-NL *Ranavirus* across tissues and that the virus first targets the oral cavity and upper respiratory mucosa. Subsequently, gastrointestinal invasion likely occurs through the vasculature as indicated by the sites of immunolabelling (connective tissues and endothelial cells). Finally, there is wide-spread involvement of numerous organs including kidneys, skin and gonads.

The ISH staining of the gastric contents shows that smooth newts in this study likely became infected by ingestion. This mode of transmission has long been recognized as highly efficient for ranaviruses^15,17,18^. The initial virus replication in the mouth region of these newts and the subsequent dissemination through connective tissues and blood vessels probably explains how the virus could avoid highly damaging physiological barriers like low gastric pH^19^.

Experimental studies with a caudate-specific *Ranavirus* (*Ambystoma tigrinum* virus) have previously shown that skin to skin contact is also a plausible route of infection in tiger salamanders (*Ambystoma tigrinum*)^17^. However, in this study, *Ranavirus* skin involvement in most *Ranavirus*-exposed animals was only evident at the latest stages of viral infection.

The efficiency of the skin barrier against *Ranavirus* transmission has been shown to depend on several factors, including the amount of antimicrobial peptides produced^20^ and the diversity of the host skin microbiome^21^. Alterations to the previous factors likely result in disruption of skin integrity and probably facilitate invasion of the pathogen and the development of disease.

Severity of disease was highest in animals infected with CMTV-NL I as seen by the type of lesions and death of some animals prior to euthanasia. These results are consistent with the previous hypothesis that the CMTV-NL I *Ranavirus* is the most pathogenic of the Dutch *Ranavirus* strains^9–11^. This likely explains the association of this strain to the on-going high mortality involving over half of the endemic amphibian species in the Netherlands, and the local declines in populations of water frogs (*Pelophylax* spp) in natural and artificial water bodies^10^. In contrast, CMTV-NL III *Ranavirus* exposed animals did not experience mortality prior to the designated euthanasia time point and even though infection was confirmed in most animals, the overall lesion severity was low. This indicates attenuated virulence of the strain, which had previously been suggested by low *Ranavirus*-associated mortality in areas where the strain occurs and by molecular data showing truncations of virulence-associated genes^11^.

Regarding *Ranavirus*-excretion by infected smooth newts, evidence of viral DNA was observed in the feces of both infected animal groups. It is possible that the initial excretion at day 2 p.i. and DNA intestinal tissue detection at 6 hours p.i., corresponded to virus inoculated during the challenge and did not necessarily reflect replication. This is supported by the positive ISH staining limited to the gastric contents at 3 days p.i. in CMTV-NL I-infected animals and the absence of IHC signal in the intestine of these animals until 9 days p.i. The number of animals with fecal viral DNA was comparable in both exposed groups. However, only CMTV NL-I *Ranavirus*-infected animals shed virus constantly until the day of euthanasia.

The results also show that *Ranavirus*-exposed smooth newts intermittently excrete virus in feces and that the virus replicates within tissues in the absence of lesions or clinical signs. This confirms that smooth newts, like other species of caudates^13^ may harbor subclinical infections and serve as natural reservoir hosts. These findings highlight the importance of increasing the scope of CMTV-NL *Ranavirus* monitoring efforts, to focus not only on sampling highly susceptible amphibian species or life stages but also amphibian accompanying species which do not show evident *Ranavirus*-associated clinical signs.

To conclude, this study signals the oral route as an effective and likely common transmission pathway of ranaviruses in smooth newts; showcases distinct pathogenicity of two CMTV-NL *Ranavirus* isolates and confirms the ability of these animals to harbor subclinical infections, excrete virus in feces and potentially serve as disease reservoirs.

## Materials and Methods

### Collection and husbandry of animals

All experimental procedures were undertaken at the Institute of Zoology (IoZ), London, UK. The husbandry methods were first approved by the Zoological Society of London Ethics Committee (project’s license number: P8897246A) and carried out in accordance to regulations.

A total of 45 adult smooth newts were collected at several natural sites in Cambridge, UK (Longitude: 52.24, Latitude:0.04) and Essex, UK (Longitude:51.58, Latitude 0.50) without a previous history of amphibian mortality or *Ranavirus* detection during the spring of 2017.

Upon arrival to the animal facility at the IoZ, newts were kept in two groups housed in plastic tanks (80 × 40 × 25 cm) filled with soil, reptile hides (Exo Terra, UK), cork bark and Petri dishes with aged tap water. In order to exclude the presence of subclinical infections, a two-month long quarantine period was implemented. Animals were monitored daily and fed a diet consisting primarily of live crickets (*Gryllodes sigillatus*) dusted with the calcium supplement (Nutrobal, US), every two days.

### Virus culture

Skin and liver from two individual *Pelophylax* spp (one from the northern province of Drenthe and another from the southern province of Limburg, NL), that were deceased and submitted to the Dutch Wildlife Health Centre (DWHC) tested positive for *Ranavirus* infection. This was confirmed by PCR targeting the *Ranavirus* major capsid protein^22^ and histopathology. Partial DNA sequencing allowed for *Ranavirus* characterization based on 7 partial genes^23^. The sequences revealed distinct CMTV-NL strains (CMTV-NL I and CMTV-NL III), and virus isolation was carried out. Remnants of frozen tissues from these two animals (skin, liver and kidney) were separately pooled, ground, and used to make 10% suspensions in L-15 growth medium (Product no. L4386-Sigma-Aldrich) containing 1% antibiotics. The suspensions were stored at 4 °C overnight, centrifuged for 10 min at 800 g at 4 °C, filtered (0.45 µm) and inoculated on confluent layers of zebra fish endothelial cells (ZENDO)^24^, supplied with L-15 growth medium. When cytopathic effect was observed, the supernatant from each animal was aliquoted and stored at −80 °C.Titrations of three aliquots were performed on 96 well plates containing ZENDO cells by evaluating tissue culture infectious dose 50% (TCID50) at 6 days post infection using the Spearman Käarber method^25^. Remaining virus aliquots from the same batch as those tittered, were shipped from the Netherlands to the UK on dry ice and immediately stored at −80 °C upon arrival.

### *In vivo* infection experiment

After the quarantine period, animals were randomized into three different groups of 15 individuals each in accordance to the three assigned treatments: Group 1: CMTV-NL I (10^8^ TCID50), Group 2: CMTV-NL III (10^8^ TCID50) and finally the control group: cell medium (L-15). These groups were further split into five subgroups of three animals each based on established post-infection (p.i.) experimental time points: 6 hours, 3, 6, 9 and 12 days p.i. Each animal was transferred to an individual plastic box using a different set of powder-free vinyl gloves. Before inoculation with the treatments, the animals were measured (snout to vent), weighed, and swabbed on the dorsal and ventral skin to exclude subclinical *Ranavirus* infections acquired in the wild. The swabs were labelled with the identification number of the animal and stored in a −80 °C freezer.

The newts were exposed percutaneously to 3 ml of *Ranavirus* suspended in cell medium (10^8^ TCID_50_) for 6 hours. The virus was pipetted directly on individual plastic trays and the newts were placed on the virus puddle to ensure full palmo-plantar and abdominal contact. These bath exposure procedures were performed under the UK Home Office license (license P8897246A), and by a trained and licensed technician. Procedures were always performed first on the control animals to avoid cross-contamination. All animals were fed every two days and their water changed daily during the experiment. Feces were collected daily when present and stored in 70% alcohol for PCR analysis.

Once each of the assigned time points was reached, the animals were humanely euthanized by trained staff using a schedule 1 method for amphibians, consisting of an overdose of MS-222 (tricaine methanesulfonate) and confirmation of death through pitting of the brain. Samples of liver, intestine and a hind-foot were collected for *Ranavirus* PCR analyses and stored in 70% alcohol. The rest of the animal was fixed in 10% formalin for 24 hours and then post-fixed in 70% alcohol.

### Histopathology and semi-quantitative scoring

All organ samples fixed in formalin were processed for routine histopathological analysis and stained with hematoxylin/eosin. To compare the pathogenicity of the two virus strains, three hallmark lesions of *Ranavirus* infection were separately and blindly evaluated in 10 organs of each individual by two veterinary pathologists using an established semi-quantitative scoring system^10^. These lesions consisted of viral inclusion bodies (scores: 0 none; 1 if <5 per high power field; 2 if 5–10 per high power field; 3 if >10 per high power field), extent of necrosis of epithelial and mesenchymal cells (scores: 0 none; 1 < 5% of organ; 2 5–50% of organ; 3 > 50% of organ), and the level of vascular damage in the form of hemorrhage, vasculitis or thrombosis (scores: 0 none; 1 mild; 2 moderate; 3 if severe). The small size of two organs (spleen and kidney) resulted in loss of the tissue from several animals during histopathology processing. These organs were scored but excluded in the final statistical analyses for lesion severity to avoid over or underestimation of the final average score per animal.

### Statistical analysis

For each treatment (a combination of both CMTV-NL *Ranavirus* strain and euthanasia day), there were three animals. Only the 10 organs available for all animals were considered for the analysis. To examine the correlation among lesions within groups, the lesion scores were analyzed with a Spearman rank correlation test, given the small sample size for each treatment at each time point (n = 3).

### *In situ* hybridization

In order to evaluate virus tissue tropism several animals from both infected groups were processed for *in situ* hybridization. Given the presumably higher sensitivity of *in situ* hybridization over immunohistochemistry, animals belonging to the first two time points (6 hours and 3 days p.i.) were selected along with animals belonging to the 9 days p.i. time point which served as a positive control. The control group was excluded based on negative PCR and immunohistochemistry results. Slides were stained with an *in situ* hybridization probe (ACBio RNAscope® Probe- V-FV3-orf90R, Cat No. 439991) which, although labeled as specific to Frog virus 3 (FV3), has been shown to target other species in the *Ranavirus* genus,. Protocol followed general manufacturers (Advanced Cell Diagnostics, 7707 Gateway Blvd. Newark, CA, 94560, USA) specifications.

### Immunohistochemistry

For immunohistochemistry, a polyclonal antibody against European catfish virus provided by Anna Toffan (Istituto Zooprofillatico Sperimentalle delle Venezie, Italy) was used. The specificity of the antibody was tested by western blot analysis. Tissues from a PCR *Ranavirus* -positive and *Ranavirus-*negative amphibians were used as positive and negative controls respectively. Details of the immunohistochemistry protocol are available elsewhere^10^.

### Ranavirus PCR

To detect viral DNA in the skin swabs and in intestine and liver, the tissues were processed for conventional PCR using diagnostic primers for the major capsid protein: Forward primer (5′-GACTTGGCCACTTATGAC-3′), reverse primer (5′-GTCTCTGGAGAAGAAGAA-3′)^22^. Primers targeting salamander β-actin: Forward (5′-TGAACCCAAAAGCCAACCGAGAAAAGAT-3′) and reverse (5′-TACGACCAGAGGCATACAGGGACAGGAC-3′) were used as an internal control^26^. DNA extraction of the skin swabs was performed using an established protocol^11^. The skin swab from a *Ranavirus* -infected smooth newt carcass from a separate unrelated experiment conducted at the IoZ was used as a positive control. Fecal samples of each newt were processed for DNA extraction as previously described^27^.

## Supplementary information

Figures S1 and S2

Supplementary Table S1. Experimental animal data

Supplementary Table S2. Spearman rank correlation coefficients for lesions per test group

Supplementary Table S3. Semiquantitative scoring results

## Data Availability

The majority of the data generated from this work is included in this paper. The rest is readily available from the corresponding author on reasonable request.
