# Supplementary material for: Common midwife toad ranaviruses replicate first in the oral cavity of smooth newts (Lissotriton vulgaris) and show distinct strain-associated pathogenicity
Source: Sci Rep. 2019 Mar 14;9:4453. doi: 10.1038/s41598-019-41214-0 (PMC6418247; doi:10.1038/s41598-019-41214-0)
Supplement: Supplementary file 1 — Figures S1 and S2 [file 41598_2019_41214_MOESM1_ESM.pdf]

# Common midwife toad ranaviruses replicate first in the oral cavity of smooth newts (*Lissotriton vulgaris*) and show distinct strain-associated pathogenicity

Bernardo Saucedo[1,2], Trenton W.J. Garner[3], Natasja Kruithof[1,2], Steven J.R. Allain[4,5], Mark J. Goodman[5], Raymond J. Cranfield[6], Chris Sergeant[3] , Diego A.Vergara[7], Marja J.L. Kik[1,2], María J. Forzán[8] , Steven J. van Beurden[1] , and Andrea Gröne[1,2$]

[1]Utrecht University, Utrecht, the Netherlands;[2] Dutch Wildlife Health Centre, Utrecht, the Netherlands,[3]Zoological Society of London, United Kingdom; [4]Durrell Institute of Ecology and Evolution, University of Kent, UK, [5]Cambridgeshire and Peterborough Amphibian and Reptile Group, United Kingdom,[6]Essex Amphibian and Reptile Group, United Kingdom,[7]National Autonomous University of Mexico, Mexico,[8]Cornell University, College of Veterinary Medicine USA.

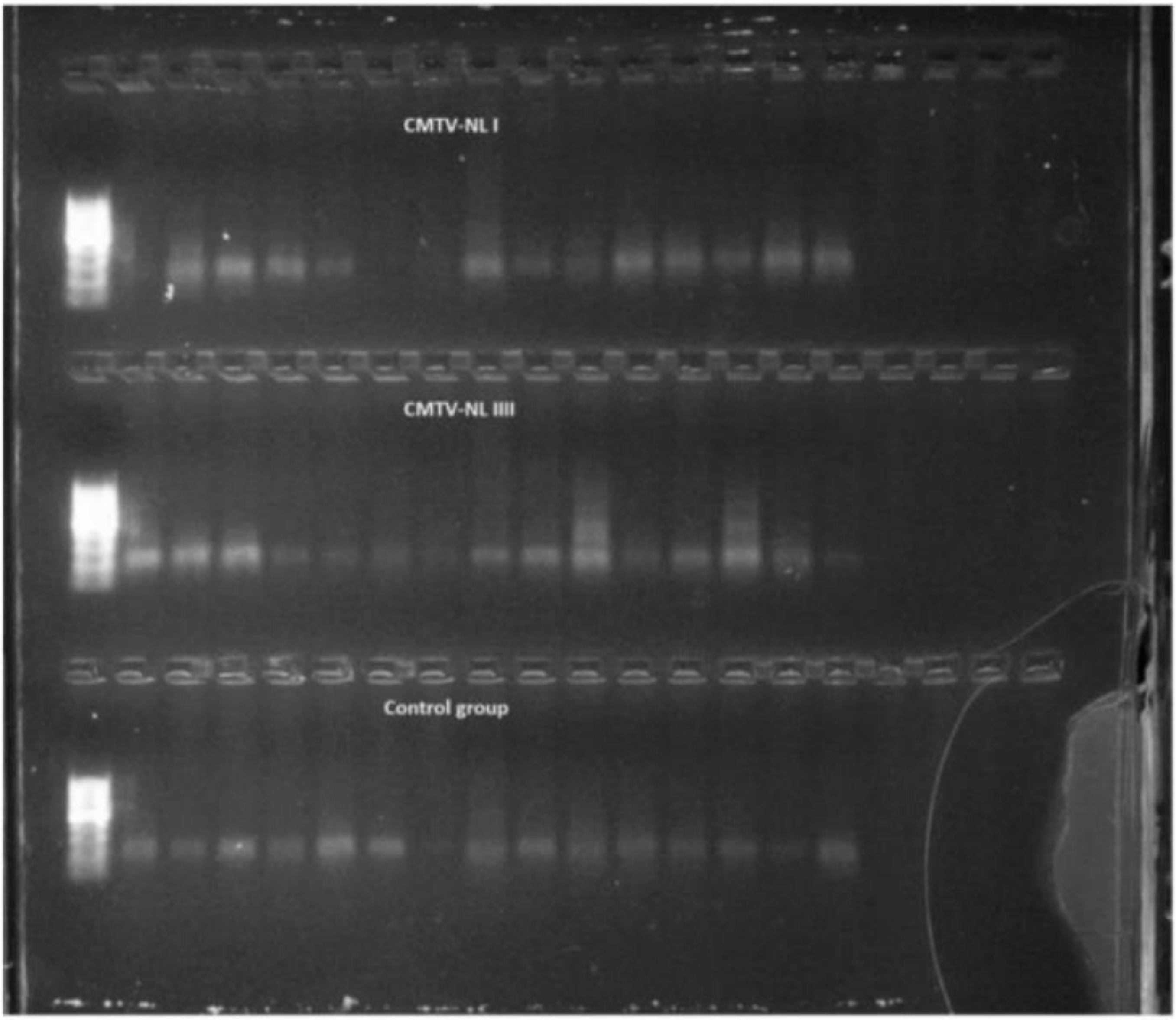


**Supplementary Figure 1**. Original gel photo from the β actin PCR of intestinal samples from the three experimental groups. Bands of varying intensity were observed in all fields

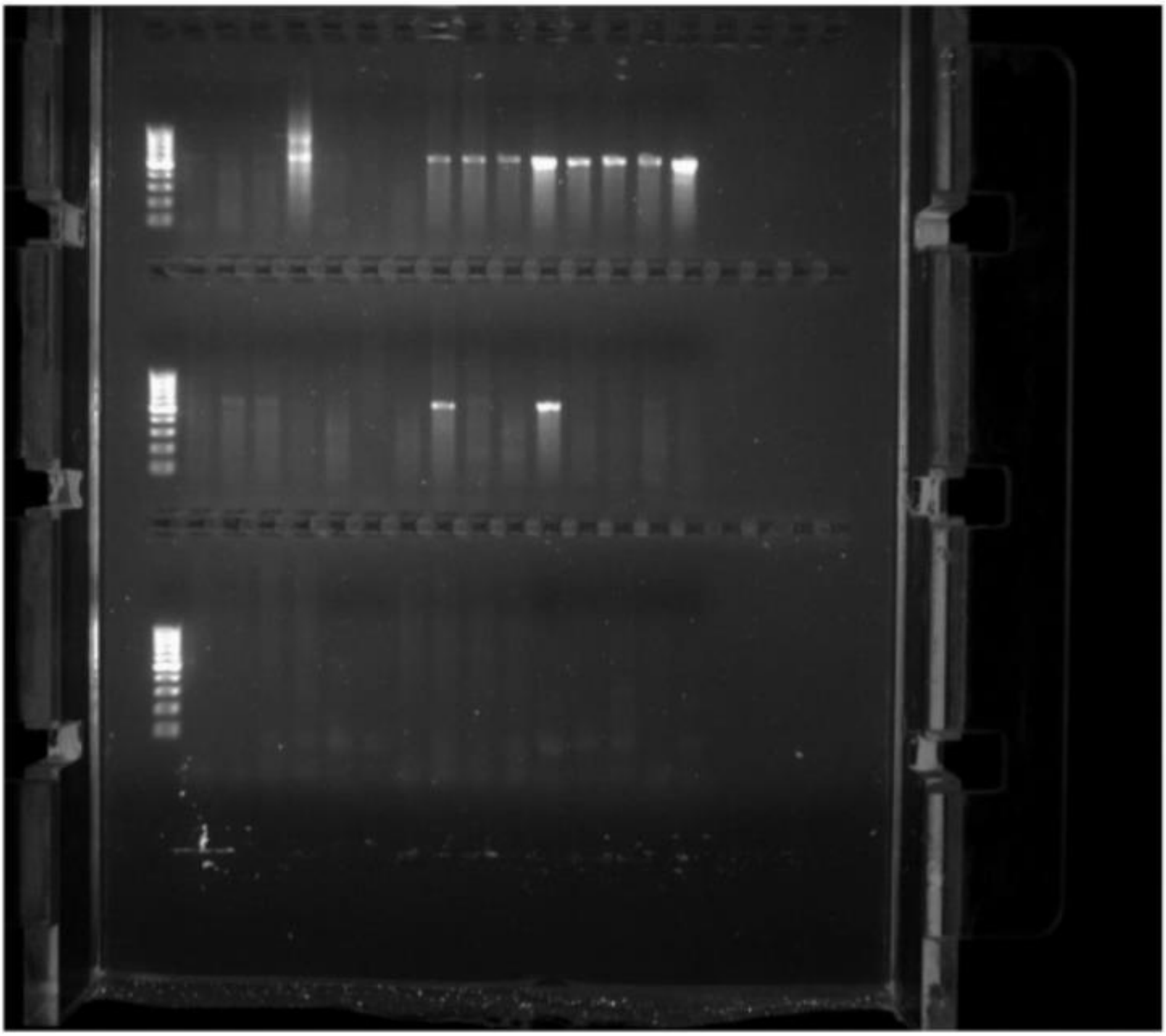

**Supplementary Figure S2.** Original gel photo from smooth newt intestinal samples tested for *Ranavirus* infection. Top Row: CMTV-NL I, medium row: CMTV-NL III, bottom row: control group.
